# Native glycan fragments detected by MALDI mass spectrometry imaging are independent prognostic factors in pancreatic ductal adenocarcinoma

**DOI:** 10.1186/s13550-021-00862-y

**Published:** 2021-12-01

**Authors:** Na Sun, Marija Trajkovic-Arsic, Fengxia Li, Yin Wu, Corinna Münch, Thomas Kunzke, Annette Feuchtinger, Katja Steiger, Anna Melissa Schlitter, Wilko Weichert, Irene Esposito, Jens T. Siveke, Axel Walch

**Affiliations:** 1 Research Unit Analytical Pathology, Helmholtz Zentrum Munich, 85764 Neuherberg, Germany; 2 Bridge Institute of Experimental Tumor Therapy, West German Cancer Center, University Hospital Essen, 45147 Essen, Germany; 3 Division of Solid Tumor Translational Oncology, German Cancer Consortium (DKTK, Partner Site Essen) and German Cancer Research Center, DKFZ, Heidelberg, Germany; 4 Institute of Pathology, TUM School of Medicine, Technical University of Munich, Munich, Germany; 5 Member of the German Cancer Consortium (DKTK), Partner Site, Munich, Germany; 6 Comprehensive Cancer Center Munich (CCCM), Munich, Germany; 7 Institute for Pathology, University Hospital Düsseldorf, Heinrich-Heine University, Düsseldorf, Germany

**Keywords:** MALDI-FT-ICR-MSI, Glycans, PDAC

## Abstract

**Background:**

Pancreatic ductal adenocarcinoma (PDAC) remains one of the deadliest malignancies to date. The impressively developed stroma that surrounds and modulates the behavior of cancer cells is one of the main factors regulating the PDAC growth, metastasis and therapy resistance. Here, we postulate that stromal and cancer cell compartments differentiate in protein/lipid glycosylation patterns and analyze differences in glycan fragments in those compartments with clinicopathologic correlates.

**Results:**

We analyzed native glycan fragments in 109 human FFPE PDAC samples using high mass resolution matrix-assisted laser desorption/ionization Fourier-transform ion cyclotron resonance mass spectrometric imaging (MALDI-FT-ICR-MSI). Our method allows detection of native glycan fragments without previous digestion with PNGase or any other biochemical reaction. With this method, 8 and 18 native glycans were identified as uniquely expressed in only stromal or only cancer cell compartment, respectively. Kaplan–Meier survival model identified glycan fragments that are expressed in cancer cell or stromal compartment and significantly associated with patient outcome. Among cancer cell region-specific glycans, 10 predicted better and 6 worse patient survival. In the stroma, 1 glycan predicted good and 4 poor patient survival. Using factor analysis as a dimension reduction method, we were able to group the identified glycans in 2 factors. Multivariate analysis revealed that these factors can be used as independent survival prognostic elements with regard to the established Union for International Cancer Control (UICC) classification both in tumor and stroma regions.

**Conclusion:**

Our method allows in situ detection of naturally occurring glycans in FFPE samples of human PDAC tissue and highlights the differences among glycans found in stromal and cancer cell compartment offering a basis for further exploration on the role of specific glycans in cancer–stroma communication.

**Supplementary Information:**

The online version contains supplementary material available at 10.1186/s13550-021-00862-y.

## Background

Pancreatic ductal adenocarcinoma (PDAC) remains one of the least understood malignancies to date, resulting in lack of targeted and immune-based approaches. Though molecular characterization and subtyping into two major subtypes [1–3], classical and basal-like/quasi-mesenchymal, have brought transcriptome-based patient stratification into focus, the underlying biology of PDAC with regard to progression and therapy resistance remains largely elusive [4]. Desmoplastic stroma that surrounds and modulates the phenotype of cancer and immune cells within the tumor microenvironment is a hallmark of PDAC and likely a direct modulator of disease progression [5]. The stroma, consisting mainly of extracellular matrix (ECM), blood vessels and ECM-producing fibroblasts, creates a setting that communicates with cancer and immune cells and modulates tumor growth, metastasis and drug resistance. Identifying molecules differentially expressed in the stroma and cancer cell compartment may allow better understanding of cancer–stroma communication and development of technologies for compartment-specific targeting and stromal modulation.

Glycans are sugar moieties, oligosaccharides or polysaccharides, that when attached to proteins and lipids create glycoconjugates. Glycans present very diverse structures, have shorter or longer, branched or linear sugar chains and are additional modified by sulfatation, phosphorylation, etc. Depending on how the sugars are attached to the protein/lipid molecule, glycoconjugates are clustered in four general groups: (i) *N*-linked glycoproteins where sugar motifs are attached to asparagine, (ii) *O*-linked glycoproteins where sugars are attached to serine/threonine, (iii) heavily glycosylated proteoglycans with one or more glycosaminoglycans (GAG) attached and (iv) glycolipids. It is estimated that 50–70% of all human proteins are post-translationally glycosylated and correct glycosylation is essential for proper cellular localization and function of the protein or lipid. Glycosylation fundamentally influences protein trafficking, stability and forming, and many proteins and lipids involved in essential physiological processes such as cell–cell communication, cell–ECM communication, protein folding and signal transduction are glycosylated. It has been shown that in tumors, including PDAC, altered glycosylation is involved in tumor-related processes of cell adhesion, proliferation, invasion, metastasis and angiogenesis [6, 7]. Typical cancer-associated glycosylation changes include sialylation, fucosylation, *O*-glycan truncation and *N*- and *O*-linked glycan branching [7], all present in PDAC as well. For example, interaction of tumor-associated sialyl Lewis antigen (SLe^*x*^) with endothelial adhesion molecules selectins allows extravasation of cancer cells and metastatic spread [8]. In PDAC, abundance of SLe^*x*^ predicts development of liver metastasis [9]. Truncated *O*-glycans have been detected on EGFR in pancreatic cancer [10], increased *N*-glycosylation and branching on integrins and ECM proteins [11], and increased fucosylation is detected in serum of PDAC patients [12]. Furthermore, the main glycan synthesis pathway, the hexosamine biosynthesis and consecutive *O*-glycosylation are strongly upregulated in highly resistant hypoxic PDAC cells [13].

Tumor stroma supports the cancer progression in multiple ways. Growth factors, adhesion molecules, nutrients are all produced by the stromal cells and locally provided to the proliferating cancer cell. There is whole plethora of interactions observed between glycosylated stromal proteins and cancer cells, all serving the function of cancer progression, invasion and metastasis (reviewed in [14]). Stromal ECM provides a settling niche for cancer and immune cells, and next to collagen fibers, glycosylated proteoglycans are the main component of tumor ECM. Proteoglycans are comprised of one or several glycosaminoglycans (GAG) with repeats of chondroitin sulfate (CS), heparan sulfate (HS) and dermatan sulfate (DS) covalently attached to protein core. Proteoglycans are mainly produced by the cancer-associated fibroblasts (CAFs) in the stroma and play a role in intercellular and ECM interactions via activation of receptor tyrosine kinases (EGFR, FGFR, IGF1R, INSR) on the cancer cells [14] regulating the proliferation and survival cascaded. Furthermore, stromal proteoglycans communicate with cancer cell membrane integrins and regulate cells motility and invading potential [14].

Due to their cancer-specific expression and function, proteins with altered glycosylation are highly attractive as potential biomarkers, targets for therapeutic agents and targeted drug delivery [15]. The diversity of glycosylated proteins and lipids and their functions in cancer opens these perspectives. However, which glycans are typically enriched in cancer cells and which specifically in the stromal compartment, respectively, has not been addressed yet. PDAC is one of the most stroma-rich solid tumors where desmoplastic reaction consumes 50–80% of the tumor tissue and can even be stratified to “normal” and “activated” stroma with later having worse prognosis [16]. Defining glycans that are differentially expressed in cancer cell or stromal compartment may improve our understanding of the stroma–cancer cell interaction and indeed allow better, compartment targeted therapeutical approaches.

In this work, we report on high mass resolution matrix-assisted laser desorption/ionization Fourier-transform ion cyclotron resonance mass spectrometric imaging (MALDI-FT-ICR-MSI)-based detection of prominent differences in native glycan fragments distribution among cancer and stromal compartments in human PDAC FFPE tissue samples. Previously performed glycan mass spectrometry studies on PDAC tissue used digestion methods for release of *N*-glycan structures from the protein core [17]. Enzymatic degradation of tissue increases measurability but hinders the differentiation among naturally occurring glycans and those previously bound [18]. Our MALDI-FT-ICR-MSI is performed on FFPE PDAC samples without previous tissues digestion with PNGase F or other biochemical process allowing detection of naturally occurring native glycan fragments, products of tissue-specific proteoglycan/GAG degradation. We classify differentially expressed glycans in cancer cells and the tumor stroma, which may have prognostic and functional relevance in PDAC progression. This glycan map may thus serve as a reference for further glycan-based diagnostic and targeting approaches.

## Methods

### Tissue microarray (TMA) preparation

Tissue microarrays (TMAs) were constructed using formalin-fixed paraffin embedded resected Pancreatic Ductal Adenocarcinoma (PDAC) specimens from 109 patients. The study was approved by the ethics committee of the Technical University of Munich, Germany (documents no. 1926/2007 and 126/2016S). Written informed consent was obtained from every patient. Representative tumor areas were identified by an experienced pathologist, and three cores with 1 mm diameter from each sample were transferred into the microarray.

### MALDI-FT-ICR MSI analysis and data processing

The FFPE samples were cut into 3 μm sections on a paraffin microtome (HM 355S, Microm, Thermo scientific), mounted onto the ITO-coated glass slides. The FFPE sections were incubated at 60 °C for 1 h, deparaffinized in xylene (2 × 8 min) and dried on a hot plate at 37 °C. The matrix solution consisted of 10 mg/ml 9-aminoacridine hydrochloride monohydrate (9-AA) (Sigma-Aldrich, Germany) in water/methanol 30:70 (*v*/*v*). SunCollect™ automatic sprayer (Sunchrom, Friedrichsdorf, Germany) was used for matrix application. The flow rates were 10, 20, 30 and 40 μl/min, respectively, for the first four layers. The other four layers were performed at 40 μl/min. The MALDI-MSI measurement was performed on a Bruker Solarix 7T Fourier-transform ion cyclotron resonance mass spectrometer (FT-ICR-MS) (Bruker Daltonik, Bremen, Germany) in negative ion mode using 50 laser shots per spot at a frequency of 500 Hz. The MALDI-MSI data were acquired over a mass range of *m*/*z* 50–1000 with 50 μm lateral resolution. The acquired data from the tissue samples were underwent spectra processing in FlexImaging v. 4.0 (Bruker Daltonics, Germany). MALDI-MSI data were normalized to the root mean square of all data points. Cancer cell and stroma regions were annotated as regions of interest (ROIs). The average spectral data of ROIs were then exported to peak list as.csv files from FlexImaging software.

MATLAB^®^ R2014b (v.7.10.0, Mathworks, Inc., Natick, MA) was used as MALDI spectral preprocessing tool for the subsequent data bioinformatics analysis. The exported.csv files including all mass spectra from cancer cell and stroma regions are loaded into MATLAB^®^ R2014b and underwent resampling, smoothing and baseline subtraction to lower the data dimensionality and to remove the noise-level peaks and artefacts. Peak picking was performed using an adapted version of the LIMPIC algorithm [19] with *m*/*z* 0.0005 minimal peak width. The signal-to-noise and intensity threshold were set to 2 and 0.01%, respectively. Isotopes were automatically identified and excluded.

### Glycan annotation

The peak list was submitted to glycan annotation using the PeakFinder tool (http://www.eurocarbdb.org/ms-tools/), which was included in GlycoWorkbench ver 2.1 build 146 (http://www.eurocarbdb.org/) [20]. The search parameters were a 4 ppm mass tolerance and a negative charge. Additionally, human Metabolome Database (http://www.hmdb.ca/) was used as supplementary database for annotation of the glycan peaks.

### Kaplan–Meier analysis and statistical analysis

In order to determine the prognostic power for each identified glycan, the individual patient glycan fragment abundances were used to split the cohort into good and poor survivor groups by the application of intensity cutoffs, which were optimized to the clinical endpoint. Cutoff-optimized survival analyses were performed as previously described [18, 21] using a Kaplan–Meier Fitter and log-rank test. Cutoff optimized in this context means that the threshold for low and high abundance of a compound was chosen such that the *p* value in the resulting Kaplan–Meier curve is minimal. Cutoff points and patients survival table are given in Additional file 2: Table S1. Overall survival rates were calculated using the Kaplan–Meier method and included 95% confidence interval estimates. Survival curves were tested with the log-rank *χ*^2^ value and Cox proportional hazards regression analysis. Univariate and multivariate analysis was performed using Cox proportional hazards regression models. In a Cox regression, the coefficients of predicate variables relate to hazard and the hazard ratio (HR) of a predicate variable is given by the exponent of its coefficient. All statistical analyses were performed within the R statistical environment (R Foundation for Statistical Computing, Vienna, Austria), in which *p* values ≤ 0.05 were considered statistically significant.

Through factor analysis, a smaller set of analyzed variables could be group into “factors” with common characteristics [22]. Factor analysis provides a way of extracting underlying “factors” or “factor constructs” which accounts for the inter-correlations among the variables [23, 24]. It serves as a good procedure to identify the factors that summarize the “good” or “poor” prognostic glycan fragments identified in MALDI-MSI analysis. Previous studies have attempted to apply similar analysis for identifying latent factors and to use these factors to stratify patients for survival analysis [25]. In this study, the analyses were done using factor analysis function (“fa”) from R “Psych” package. Default factoring method (“minres”) and rotating method (“oblimin”) were applied. To explore the optimal factor numbers to extract, fa.parallel function was applied for the “screen plot” which helps determining the factor numbers to extract. Factor analysis generates factor scores for each unraveled factor. As a summarization of the variation of a set of related original variables, factor score represents the variation of extracted “factor” variable among samples and can be used in subsequent analyses [26]. In this study, factor scores are applied in Kaplan–Meier analysis and multivariate survival analysis.

## Results

### Patient characteristics and data acquisition

The MALDI-FT-ICR MSI analysis included 109 PDAC patients. Detailed clinical characteristics of PDAC patients are shown in Table 1. Within the mass range of *m*/*z* 50 to 1000, approximately 2000 individual MS peaks per pixel could be resolved. With 50 μm lateral resolution, the MSI spectra data of more than one million pixels were extracted and subjected to metabolic database and bioinformatics analysis.

**Table 1:** Clinical characteristics of the PDAC patients

Characteristic	Number (%) of patients or median (range)-tumor regions
Number of patients	109
Male Sex; number (%)	60 (55)
Age at initial diagnosis (years)	69 (32–87)
Survival time (months)	17 (3–69)
Status complete	84 (77)
UICC tumor stage at initial diagnosis; number (%)	
I	5 (5)
II	83 (76)
III	13 (12)
IV	8 (7)

### Mass spectrometry imaging reveals differences in glycan distribution in tumor and stroma regions

**Fig. 1:**
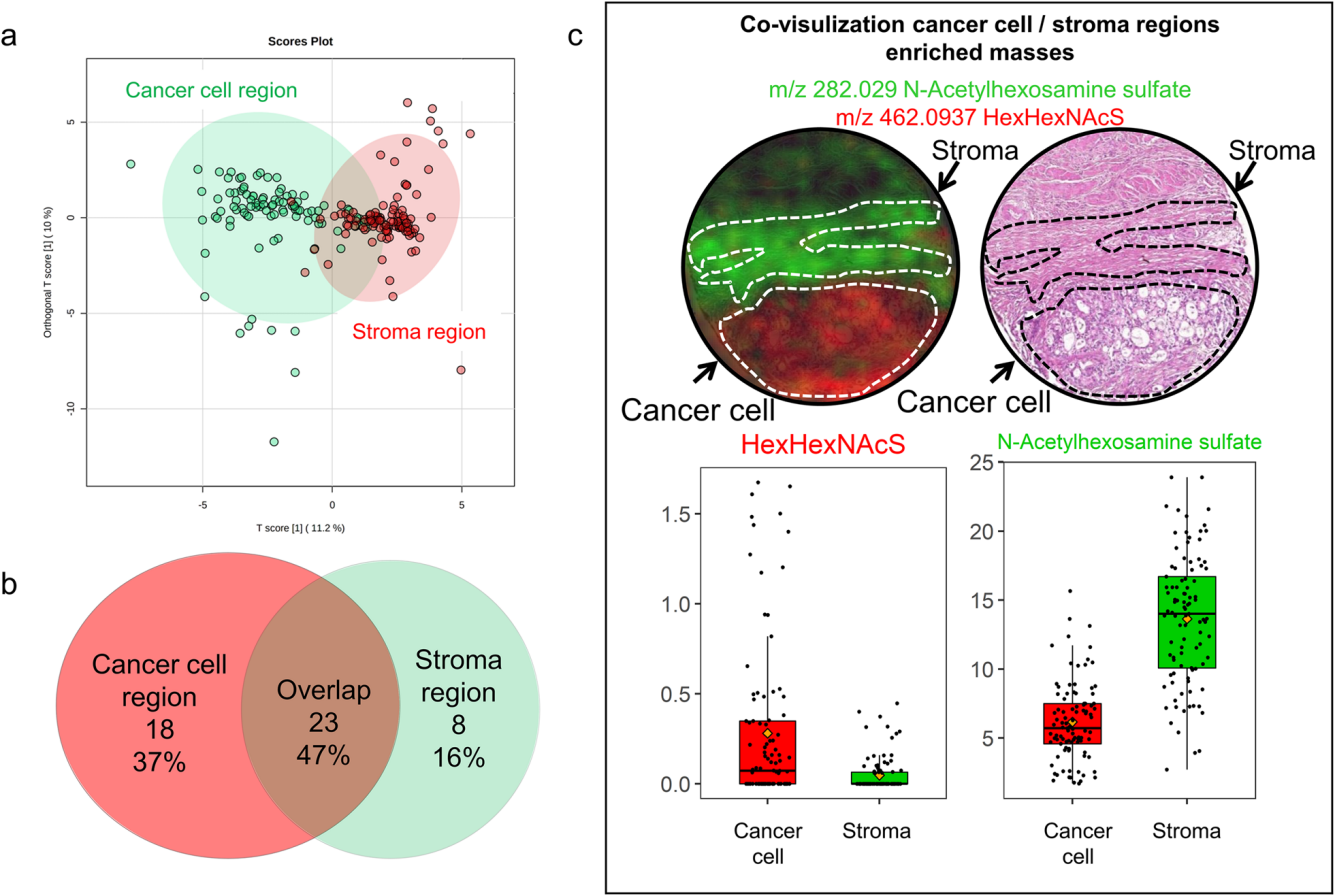
**a** Ortho-PLSDA of cancer cell and stroma region based on glycan intensities. **b** Venn diagram indicating common and distinct glycan fragments detected in cancer cell and stroma regions, respectively. **c** Example of glycan fragments differently expressed in tumor and stroma region, respectively. Co-visualization of HexHexNAcS (red color) and *N*-Acetylhexosamine sulfate (green color) representing different distributions in the cancer cell and stroma regions

PDAC tissue cores were investigated for regions rich in cancer cells (hereafter always named as “cancer cell region”) and stroma region and were manually annotated. For glycan annotation, GlycoWorkbench and HMDB database were used. As a result, 41 native glycan fragments were detected in cancer cell regions, and 31 glycan fragments were identified in the stroma region. Both unique and common glycans could be detected in cancer cell and stromal regions. The Venn diagrams show 23 glycans commonly identified in cancer cell and stroma regions (Fig. 1B). List of glycan fragments and their localization are summarized in Table 2. Additionally, differences among glycan fragments found in cancer cell and stroma region could be observed as indicated by orthogonal partial least squares discriminant analysis (ortho-PLSDA) (Fig. 1A). As an example, in Fig. 1C two differentially distributed glycan fragments in one tissue core are co-visualized, showing a distinct enrichment of Hex-HexNAcS in the cancer cell region and *N*-Acetylhexosamine sulfate in the stroma region.

**Table 2:** List of annotated glycan fragments

Annotation	MW	Cancer cell and/or stroma region
HexA	193.0350	Cancer cell/stroma
*N*-Acetylhexosamine	202.0720	Cancer cell/stroma
HexS	259.0135	Cancer cell/stroma
HexP	259.0225	Cancer cell/stroma
*N*-Acetylhexosamine sulfate	282.0290	Cancer cell/stroma
HexHex	341.1090	Cancer cell/stroma
NeuGcAc	366.1030	Cancer cell/stroma
Chondroitin or hyaluronan	378.1050	Cancer cell/stroma
dHexHexANAcMe	394.1360	Cancer cell/stroma
HexAHexNAc	396.1157	Cancer cell/stroma
*N*-Acetylhexosamine disulfate	401.9785	Cancer cell/stroma
HexNS3	417.9440	Cancer cell/stroma
HexNAcHexNAc	423.1620	Cancer cell/stroma
HexHexNAcAc	424.1463	Cancer cell/stroma
HexANAcHexNAc	437.1397	Cancer cell/stroma
Chondroitin sulfate	458.0605	Cancer cell/stroma
HexHexNAcS	462.0937	Cancer cell/stroma
HexAHexNAcS	476.0725	Cancer cell/stroma
dHexPenHexAc	499.1677	Cancer cell/stroma
dHexHexHexAMe or Hex2PenAc	515.1625	Cancer cell/stroma
PenHexAHexNAc	528.1555	Cancer cell/stroma
HexNAcHexAHexNAc or NeuAcAc	599.1955	Cancer cell/stroma
PenPenHexAMePenHexAMe	793.2255	Cancer cell/stroma
dHexHexN	324.1300	Cancer cell
HexNAcAcAcAc	346.1140	Cancer cell
NeuAcAc	350.1090	Cancer cell
dHexHexS	405.0710	Cancer cell
HexNAcHexNAcS	503.1175	Cancer cell
PenPenHexAcAc	527.1625	Cancer cell
HexAHexAHexMeMe or HexHexHexAAc	559.1510	Cancer cell
HexHexHexS	583.1190	Cancer cell
KdoHexAHexA	589.1270	Cancer cell
HexHexHexNAcS	624.1455	Cancer cell
HexHexNAcHexNAcS	665.1725	Cancer cell
HexHexHexHexAc or PenPenPenPenHex	707.2260	Cancer cell
HexNAcPHexNeuAc	753.1975	Cancer cell
dHexHexHexSHexNAc	770.2043	Cancer cell
HexHexHexNAcHexNAcS	827.2250	Cancer cell
HexHexPenHexAHexNAc	852.2610	Cancer cell
HexHexHexHexHexAc	869.2800	Cancer cell
dHexdHexPenPenHexHex	897.3110	Cancer cell
PenHexNAc	352.1250	Stroma
HexMeHexNAc	396.1500	Stroma
dHexHexNAcS	446.0990	Stroma
HexHexHexMe	517.1790	Stroma
HexNAcdHexdHexPen	644.2397	Stroma
dHexdHexdHexHexAMe or dHexdHexHexPenAc	645.2233	Stroma
PenPenHexdHexSMe	683.1730	Stroma
HexNAcHexNeuAcMeMe	701.2640	Stroma

*Hex* hexose, *dHex* deoxy hexose, *Pen* pentose, *HexA* hexuronic acid, *HexN* hexosamine, *HexNAc N*-acetylhexosamine, *NeuAc N*-acetyl neuraminic acid, *NeuGc N*-glycolyl neuraminic acid, *S* sulfate, *P* phosphate, *Ac* acetate; *NAc N*-acetate, *Me* methyl

In addition, of all identified masses, intensities for 3 glycan fragments (HexHexHexNAcS, HexHexHexNAcHexNAcS and HexHexNAcS) were significantly correlating with tumor grade (G1–G3 grades), with usually highest intensities measured in grade 1 PDACs (Additional file 1: Fig. S1).

### Impact of native glycan fragments on prognosis

To determine whether glycans expressed in cancer cell or stroma region are associated with clinical outcome, we performed Kaplan–Meier analyses with overall survival as endpoint.

**Fig. 2:**
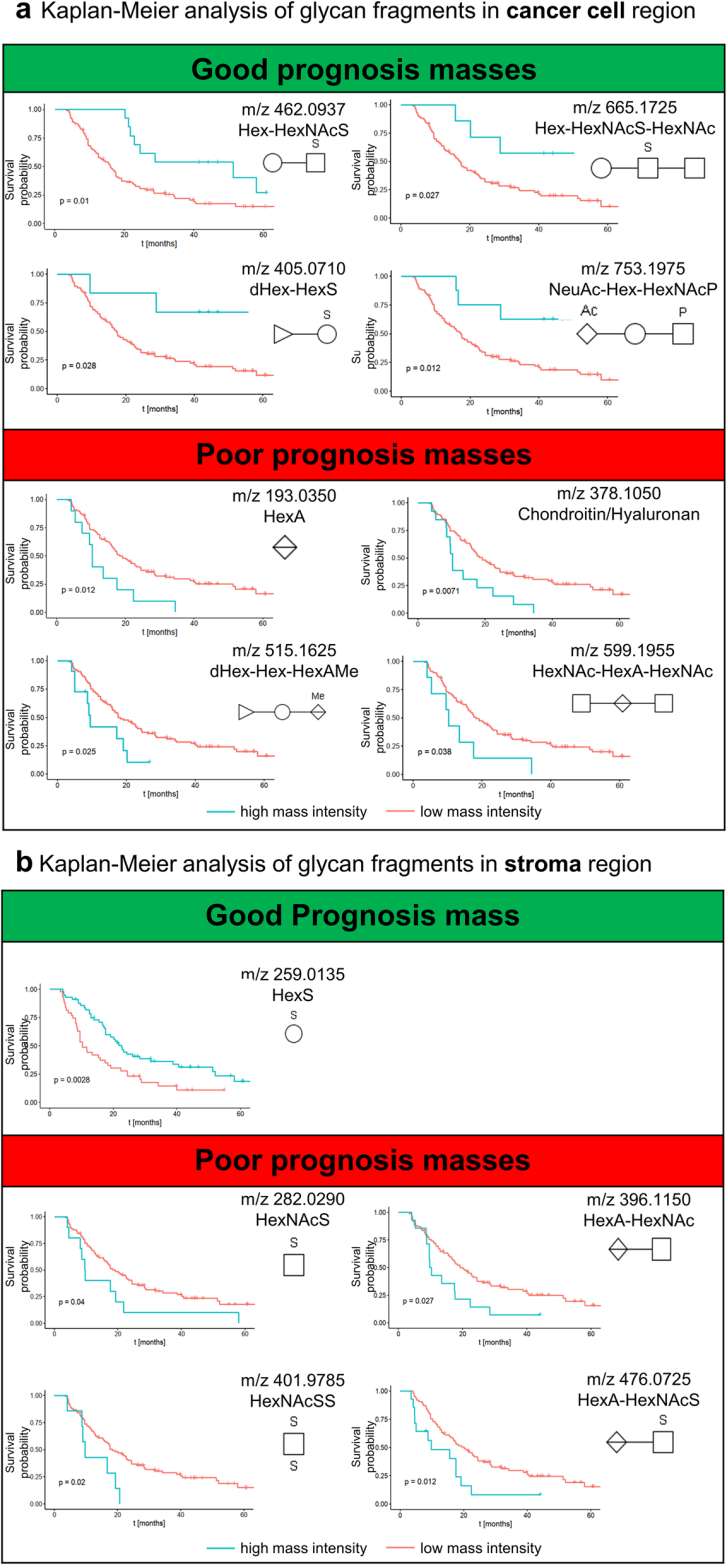
**a** Kaplan–Meier analysis of glycan fragments in cancer region. Blue lines indicate survival in patients with high intensity of the respective mass. Red lines indicate survival in patients with low intensity of the respective mass. Example Kaplan–Meier curves of 4 good prognosis and 4 poor prognosis glycan masses in cancer cell region are shown. In the upper panel, a high abundance of Hex-HexNAcS (*m*/*z* 462.0937), Hex-HexNAcS-HexNAc (*m*/*z* 665.1725), dHex-HexS (*m*/*z* 405.0710) and NeuAc-Hex-HexNAcP (*m*/*z* 753.1975) in cancer cell regions was associated with good prognosis. In contrast, in lower panel, high abundance of HexA (*m*/*z* 193.0350), chondroitin/hyaluronan (*m*/*z* 378.1050), dHex-Hex-HexAMe (*m*/*z* 515.1625) and (*m*/*z* 599.1955) HexNAc-HexA-HexNAc was associated with poor patient prognosis. **b** Kaplan–Meier analysis of glycan fragments in the stroma region. Blue lines indicate survival in patients with high intensity of the respective mass. Red lines indicate survival in patients with low intensity of the respective mass. High abundance of HexS (*m*/*z* 259.0135) in stroma regions was associated with good prognosis. High abundance of HexNAcS (*m*/*z* 282.0290), HexA-HexNAc (*m*/*z* 396.1150), HexNAcSS (*m*/*z* 401.9785) and HexA-HexNAcS (*m*/*z* 476.0725) in stroma regions was associated with poor prognosis

In the cancer cell region, Kaplan–Meier survival model identified 16 glycan fragments to be significantly associated with patient outcome (Fig. 2A, Table 3). Ten glycan fragments presented hazard ratio (HR) lower than 1 (HR < 1; 95% confidence interval (CI)), indicating favored prognosis for patients with high abundance of these glycans in the cancer cell region. The remaining 6 glycan fragments showed a HR > 1 (95% CI), indicating poor prognosis for patients with high abundance of these fragments.

**Table 3:** Univariate cox proportional hazard modeling (K–M significant)

Glycan fragments	*p* value (log *p*)	HR (95% CI)
Cancer cell region		
HexS	0.023*	0.47 (0.24–0.91)
dHexHexS	0.028*	0.24 (0.058–0.96)
HexHexNAcAc	0.038*	0.47 (0.22–0.97)
HexHexNAcS	0.01*	0.39 (0.19–0.82)
dHexPenHexAc	0.027*	0.60 (0.38–0.95)
KdoHexAHexA	0.011*	0.29 (0.11–0.80)
HexHexHexNAcS	0.023*	0.39 (0.17–0.91)
HexHexNAcHexNAcS	0.027*	0.30 (0.092–0.93)
HexNAcHexPNeuAc	0.012*	0.25 (0.079–0.80)
dHexHexHexSHexNAc	0.011*	0.25 (0.079–0.80)
HexA	0.012*	2.32 (1.19–4.53)
Chondroitin or hyaluronan	0.007**	2.25 (1.23–4.10)
HexAHexNAc	0.007**	2.25 (1.23–4.10)
Chondroitin sulfate	0.026*	1.74 (1.06–2.85)
dHexHexHexAMe	0.025*	2.19 (1.08–4.44)
HexAHexNAcHexNAc	0.038*	2.25 (1.03–4.92)
Stroma region		
HexS	0.0028**	0.50 (0.32–0.80)
*N*-Acetylhexosamine sulfate	0.040*	2.00 (1.02–3.91)
HexAHexNAc	0.027*	1.97 (1.07–3.61)
*N*-Acetylhexosamine disulfate	0.020*	2.49 (1.12–5.52)
HexANAcS	0.012*	2.19 (1.17–4.09)

"*" p<0.05; "**" p<0.01

In the stroma region, 5 glycan fragments with potentially prognostic value were identified (Fig. 2B, Table 3). High abundance of HexS was associated with good patient outcome (HR = 0.50; 95% CI). Increased intensity of the remaining four molecules corresponded to an unfavorable patient prognosis (HR > 1; 95% CI).

In addition, we also performed a multivariate analysis of the identified “prognosis significant” glycan fragments with UICC staging (Additional file 3: Table S2). Four glycan masses, dHexPenHexAc and dHexHexHexAMe in cancer cell region, HexS and HexANAcS in stroma region, served as an independent prognostic factor with regard to the Union for International Cancer Control (UICC) stage.

### Factor analysis

**Fig. 3:**
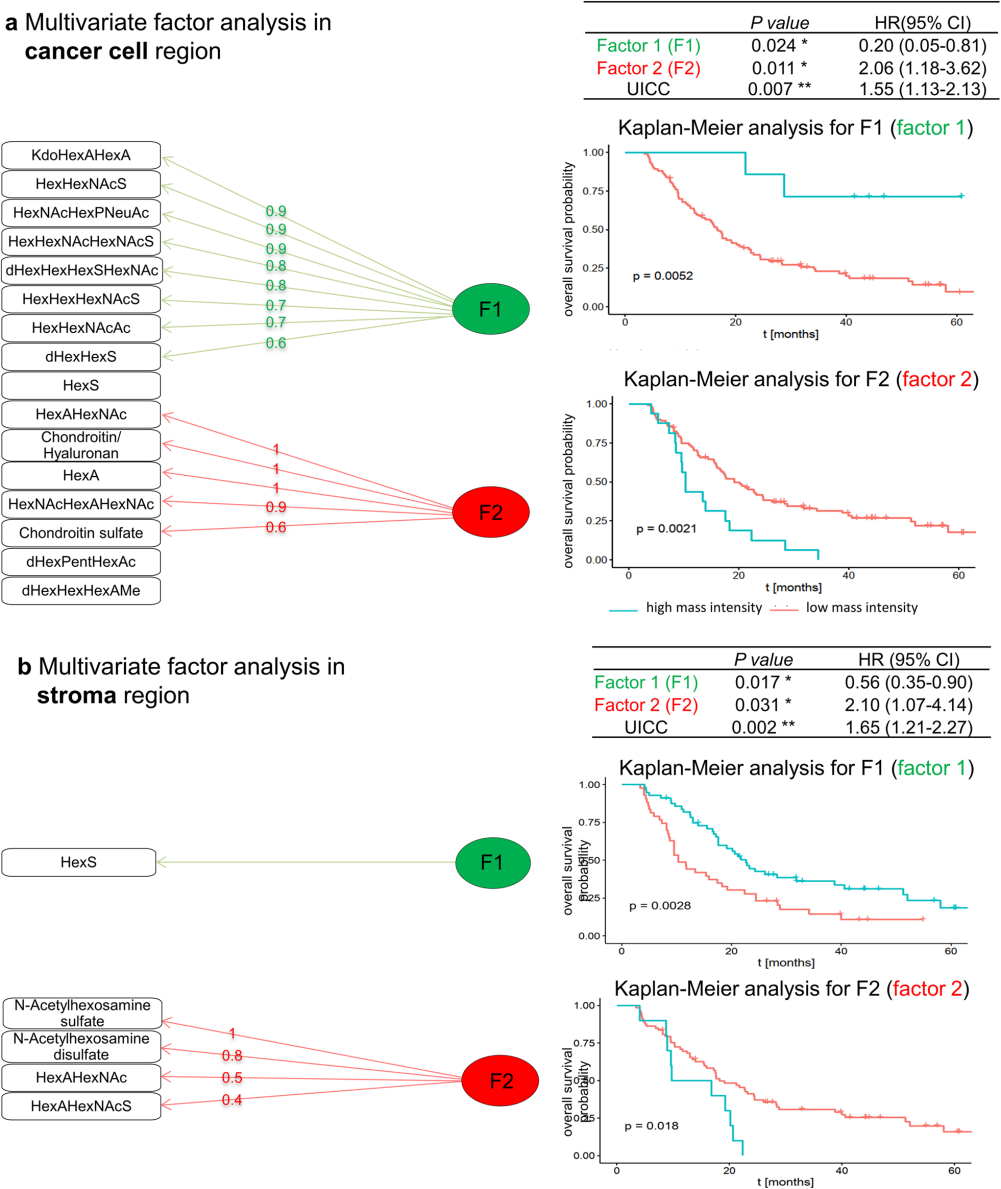
**a** Multivariate factor analysis of significant glycan fragments identifies prognostic glycan factors in cancer cell region. **b** Multivariate factor analysis of significant glycan fragments identifies prognostic glycan factors in stromal region. The numbers on the arrows pointing from the factors to the individual glycan fragments represent the factor loading of each individual glycan fragment on that factor, which quantifies the extent to which the glycan fragment is related to a given factor. Values close to 1 or − 1 represent strong relation, while values close to 0 indicate weak relation. Blue line indicates high score of the factor. Red line indicates low score of the factor

To generate independent glycan prognostic factor, factor analysis as a dimension reduction method was applied. This procedure analyzes the structure of a dataset by identifying the interrelationships among a set of various observed variables. In the cancer cell region, a clear separation of “good” prognosis fragments from “poor” prognosis fragments is achieved in the 2-factor result. Eight of 10 glycan fragments positively associated with favored patient outcome are grouped in Factor 1, while 5 of 6 fragments positively associated with poor patient outcome are grouped in Factor 2 (Fig. 3A).

In the stroma region, the glycan fragment HexS was associated with favorable patient outcome. The other 4 glycan fragments were grouped as “poor” prognosis factor (Fig. 3B).

### Multivariate analysis reveals distinct glycan patterns as independent prognostic factors

In this study, generated factor scores were applied in a multivariate analysis. In this way, the adverse influence of multiple inter-correlations among the glycans on multivariate survival modeling is mitigated. Multivariate survival model for the tumor regions integrated UICC tumor stage with Factor 1 and Factor 2. The result revealed that in cancer cell region, both Factor 1 (*p* = 0.024, HR = 0.20) and Factor 2 (*p* = 0.011, HR = 2.06) serve as independent prognostic factor with regard to the established UICC classification (*p* = 0.007, HR = 1.55) (Table 4). For the stroma region, multivariate model was applied to UICC tumor stage, the good prognoses Factor 1 and poor prognoses Factor 2. The result demonstrated the independent prognostic power of Factor 1 (*p* = 0.017, HR = 0.56) and Factor 2 (*p* = 0.031, HR = 2.10) with regard to UICC staging (*p* = 0.002, HR = 1.65) in the stroma region (Table 4).

**Table 4:** Multivariate cox proportional hazard modeling

	*p* value	HR(95% CI)
Cancer cell region		
Factor 1	0.024*	0.20 (0.05–0.81)
Factor 2	0.011*	2.06 (1.18–3.62)
UICC	0.007**	1.55 (1.13–2.13)
Stroma region		
Factor 1	0.017*	0.56 (0.35–0.90)
Factor 2	0.031*	2.10 (1.07–4.14)
UICC	0.002**	1.65 (1.21–2.27)

"*" p<0.05; "**" p<0.01

## Discussion

Considering the late diagnosis, high intrinsic and acquired resistance to chemotherapies and the resulting high mortality in PDAC, identification of early disease biomarkers and novel targets are a priority task to improve clinical outcome. Recent studies suggest that the heavily developed tumor stroma in PDAC is actively participating in PDAC biology and influences the clinical outcome [16]. Particularly cancer-associated fibroblasts (CAFs) not only produce the ECM but actively support inflammatory and migratory actions of cancer and immune cells [27]. Knowledge about molecules specifically expressed on stromal and cancer cell compartment may allow development of strategies for disruption of cancer cell–stroma synergy and provide clinical improvement. Protein and lipid glycosylation is prominently altered in cancer [7]. Carbohydrate antigen CA19-9, the only clinically relevant PDAC biomarker used for therapy follow-up, is a tetrasaccharide cleaved from membrane glycoproteins of epithelial cancer cells and released into the blood. Very recently, a prominent study with targeted CA19-9 overexpression in pancreas of genetically engineered mice suggested not only a biomarker but also a functional role of tissue CA19-9 in progression of pancreatitis to PDAC and highlighted CA19-9 as a therapeutic target [28].

To date, most of glycan research performed in PDAC focuses on serum biomarkers and specificities in PDAC tissue glycosylation patterns have been rarely addressed. Latest work showed that C1GALT1 controlled glycosylation of cell surface integrins in PDAC cells leads to increased invasiveness and poor patient survival [29] and a very recent MALDI-IMS study performed on formalin-fixed human PDAC FFPE specimens reports on distinct *N*-glycan populations presented in healthy pancreas and PDAC tissue [17] with high sialylation, poly-LacNAc extensions and fucosylation of high mass *N*-glycans specifically detected in PDAC. Due to high abundance and functional significance of stroma in PDAC, we postulated that different glycoproteins, GAGs and proteoglycans may be present in the two compartments, cancer cells and stroma, and their biological degradation may generate different glycan fragments. Identification of those fragments may help in addressing the biological differences and functionality of the two compartments.

We used a MALDI-MSI approach on a collection of 109 PDAC patient FFPE samples where the respective stromal and cancer cell compartment was annotated and separately analyzed. Differently than in mentioned recent studies where tissue digestion was used for release on *N*-glycan bound sugars [17], we used an MALDI-FT-ICR MSI with high mass resolution and accuracy that does not demand tissue pre-treatment. This method was previously optimized by our group [18, 30] and allows detection of naturally occurring unbound, native glycans in FFPE fixed tissues. We identified 8 and 18 glycan fragments uniquely expressed in only stromal or only cancer cell compartment in PDAC, respectively. In both compartments, specific glycans predicting worse or better patient survival were identified, suggesting functional implications of the identified glycans in cancer. It is, however, still difficult to interpret the origin of the identified glycan fragments as they can be biodegradation products of multiple complex proteoglycans and glycosaminoglycans (GAG). Hyaluronan and chondroitin sulfate are GAGs that show 12- and 22-fold increase, respectively, in PDAC in comparison with normal pancreas [31]. Hydrolytic cleavage of hyaluronan yields HexA-HexNAc and HexNAc-HexA-HexNAc fragments [18]. High intensity of those fragments in stroma and tumor, respectively, predicted worse survival in our study, going along with the current view that hyaluronan maintains high interstitial pressure in PDAC stroma, thus hampers the delivery of drugs and imposes lower therapy response and worse prognosis [32]. Enzymatic digestion of stromal hyaluronan with hyaluronidases improved delivery of chemotherapeutic drug Gemcitabine into the murine PDAC and prolonged survival [32, 33]. It should also be noted that hyaluronan and chondroitin masses cannot be distinguished at the full mass mode used here. Thus, the mass identified as hyaluronan may as well be chondroitin and must be carefully interpreted.

Chondroitin sulfate (CS) is not only very abundant GAG in the PDAC but also shows alterations in the sulfatation pattern of disaccharide chains with dramatically less abundant 4-sulfated disaccharides and more abundant non-sulfated disaccharides than in normal pancreas [31]. Hydrolytic cleavage of CS yields HexS, HexNAcS, HexA–HexNAc, HexA-HexNAcS and HexNAc-HexA-HexNAc fragment signals [18], and these fragments have different predictive values in PDAC tumor and stromal regions. High abundance of HexS in stroma predicts better survival, while HexNAcS and HexAHexNAcS abundance predicts worse survival. In the cancer cell compartment, chondroitin abundance predicts poor survival. At this point, it is difficult to explain those differences, but they might indicate differential activities of sulfatases and sulfotransferases that are responsible for addition and removal of sulfate groups from saccharides creating differently abundant sulfated and non-sulfated glycan fragments in cancer cell and stromal compartment.

It is also important mentioning that we detected Kdo glycan fragments in the cancer cell compartment. Kdo glycan fragments are parts of lipopolysaccharide (LPS) in the outer cell membrane of gram negative bacteria. Recent publications support the existence of intratumoral microbiome in PDAC and even suggest active influence of this microbiome on tumor growth and immune infiltration finally resulting in patient survival differences [34]. The Kdo fragments identified here may originate from the gut microbiota since the gut is in the direct vicinity of the pancreas and there may be surgical cross-contamination between gut and PDAC during biopsy but may also originate from the tumor-specific microbiota. It is tempting to speculate that MALDI-based identification of Kdo fragments may be used for defining the specific intratumoral microbiota. This, however, remains to be addressed in the future studies.

Our multivariate analysis incorporating UICC classification demonstrated that distinct glycan patterns are independent prognostic factors for PDAC patients. Thus, the identified factors and glycan groups may potentially serve clinical prediction purposes. Glycosylated proteins located on the extracellular side of the plasma membrane coordinate the interplay between the cancer cells and the ECM in the tumor microenvironment [14]. Considering the limited number of stroma/cancer cells differentiating glycans determined in our study, these glycans might serve as starting point for further functional analysis.

## Conclusions

With our approach, we provide insights into general glycome differences among the stromal and cancer cell compartment in human PDAC. Our approach using FFPE samples for MALDI-MSI offers a basis for further exploration on role of specific glycans in cancer cell–stroma communication.

## Supplementary Information

**Additional file 1. Supplementary Figure 1:** Correlation of glycan mass intensities and tumor grading (G). All 3 glycans are most abundant in G1 PDACs. Columns indicate mean+standard error. P values calculated with the Kruskal-Wallis test. *P<0.05; **P<0.01; ***P<0.001.

**Additional file 2. Supplemetary Table 1:** Cut-off points and patients survival table of Kaplan-Meier analysis.

**Additional file 3. Supplementary Table 2:** Multivariate analysis of the prognosis significant glycan fragments with Union for International Cancer Control (UICC) stage. HR-hazard ratio; CI-confidence interval.

## Data Availability

All data presented in this study are available from the corresponding authors upon a reasonable request.

## Abbreviations

PDAC: Pancreatic ductal adenocarcinoma
MALDI-FT-ICR-MSI: Matrix-assisted laser desorption/ionization Fourier-transform ion cyclotron resonance mass spectrometric imaging
UICC: Union for International Cancer Control
ECM: Extracellular matrix
TMA: Tissue microarray
FFPE: Formalin-fixed paraffin embedded
