# Supplementary material for: Native glycan fragments detected by MALDI mass spectrometry imaging are independent prognostic factors in pancreatic ductal adenocarcinoma
Source: EJNMMI Res. 2021 Dec 1;11:120. doi: 10.1186/s13550-021-00862-y (PMC8636555; doi:10.1186/s13550-021-00862-y)
Supplement: Supplementary file 1 — Additional file 1. Supplementary Figure 1: Correlation of glycan mass intensities and tumor grading (G). All 3 glycans are most abundant in G1 PDACs. Columns indicate mean+standard error. P values calculated with the Kruskal-Wallis test. *P<0.05; **P<0.01; ***P<0.001. [file 13550_2021_862_MOESM1_ESM.docx]

Supplementary figure 1:


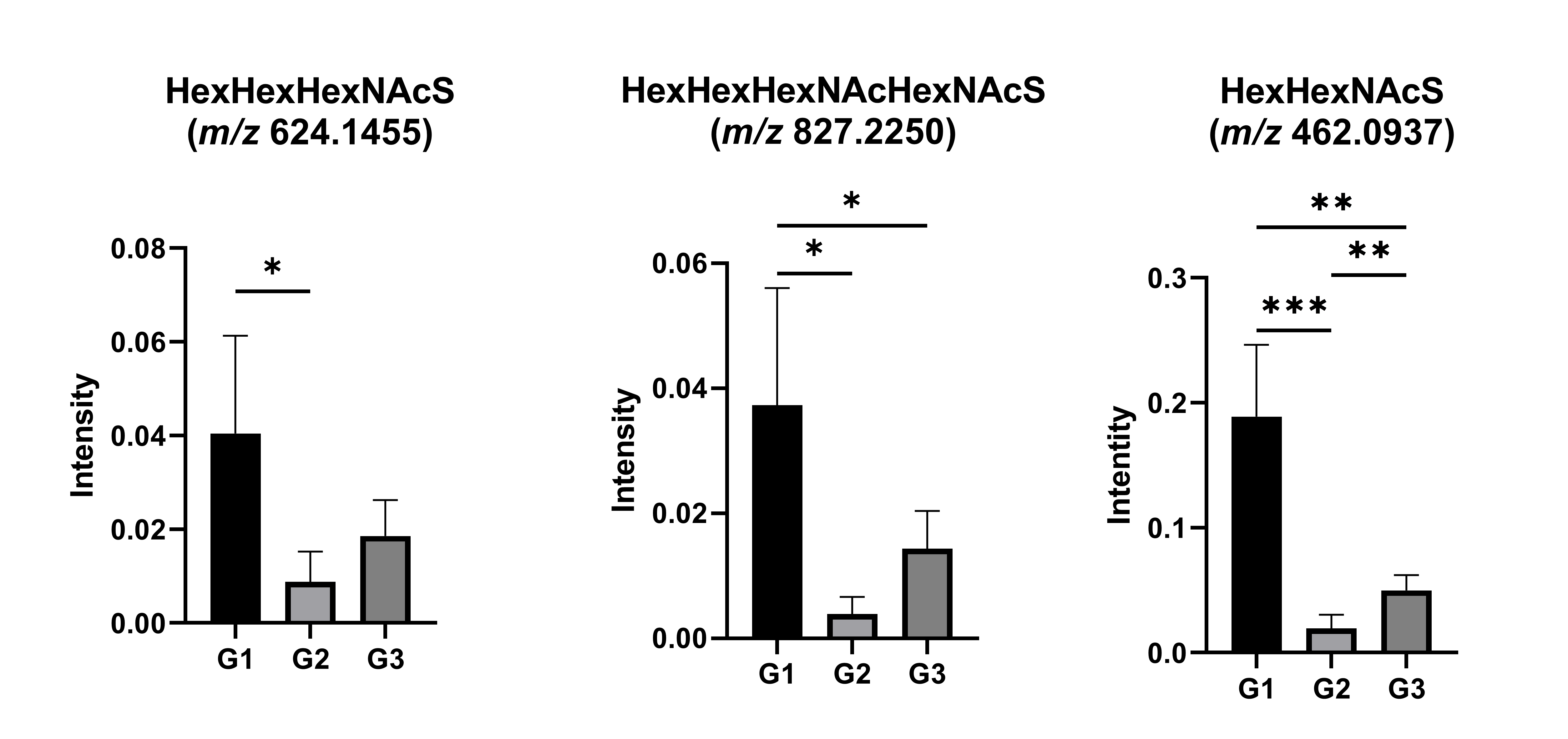


**Supplementary figure 1:** Correlation of glycan mass intensities and tumor grading (G). All 3 glycans are most abundant in G1 PDACs. Columns indicate mean+standard error. P values calculated with the Kruskal–Wallis test. * P<0.05; **P<0.01; ***P<0.001.
