# Supplementary material for: Native glycan fragments detected by MALDI mass spectrometry imaging are independent prognostic factors in pancreatic ductal adenocarcinoma
Source: EJNMMI Res. 2021 Dec 1;11:120. doi: 10.1186/s13550-021-00862-y (PMC8636555; doi:10.1186/s13550-021-00862-y)
Supplement: Supplementary file 2 — Additional file 2. Supplemetary Table 1: Cut-off points and patients survival table of Kaplan-Meier analysis. [file 13550_2021_862_MOESM2_ESM.docx]

Supplementary Table 1:

| **Cancer cell region** | | | | | | | |
| --- | --- | --- | --- | --- | --- | --- | --- |
|  | | | | survived patient at each time point | | | |
| m/z | annotation | cut-off intensity | range | 0 month | 20 month | 40 month | 60 month |
| 259.0135 | HexS | 0.331 | < 0.331 | 89 | 34 | 12 | 3 |
|  |  |  | > 0.331 | 18 | 13 | 6 | 1 |
| 405.0710 | dHexHexS | 0.0508 | < 0.0508 | 101 | 42 | 14 | 3 |
|  |  |  | > 0.0508 | 6 | 5 | 4 | 1 |
| 424.1463 | HexHexNAcAc | 0.376 | < 0.376 | 93 | 36 | 13 | 2 |
|  |  |  | > 0.376 | 14 | 11 | 5 | 2 |
| 462.0937 | HexHexNAcS | 0.819 | < 0.819 | 94 | 34 | 22 | 2 |
|  |  |  | > 0.819 | 13 | 13 | 7 | 2 |
| 499.1677 | dHexPentHexAc | 1.61 | < 1.61 | 31 | 9 | 3 | 0 |
|  |  |  | > 1.61 | 76 | 38 | 15 | 4 |
| 624.1455 | HexHexHexNAcS | 0.089 | < 0.089 | 97 | 37 | 13 | 2 |
|  |  |  | > 0.089 | 10 | 10 | 5 | 2 |
| 665.1725 | HexHexNAcHexNAcS | 0.113 | < 0.113 | 100 | 41 | 14 | 2 |
|  |  |  | > 0.113 | 7 | 6 | 4 | 2 |
| 753.1975 | HexNAcPHexNeuAc | 0.0825 | < 0.0825 | 99 | 41 | 13 | 2 |
|  |  |  | > 0.0825 | 8 | 6 | 5 | 2 |
| 770.2043 | dHexHexHexSHexNAc | 0.0868 | < 0.0868 | 99 | 40 | 13 | 2 |
|  |  |  | > 0.0868 | 8 | 7 | 5 | 2 |
| 193.0350 | HexA | 0.151 | < 0.151 | 97 | 45 | 18 | 4 |
|  |  |  | > 0.151 | 10 | 2 | 0 | 0 |
| 378.1050 | Chondroitin/Hyaluronan | 0.303 | < 0.303 | 94 | 44 | 18 | 4 |
|  |  |  | > 0.303 | 13 | 3 | 0 | 0 |
| 396.1157 | HexAHexNAc | 0.666 | < 0.666 | 94 | 44 | 18 | 4 |
|  |  |  | > 0.666 | 13 | 3 | 0 | 0 |
| 458.0605 | Chondroitin sulfate | 0.891 | < 0.891 | 80 | 41 | 15 | 3 |
|  |  |  | > 0.891 | 27 | 6 | 3 | 1 |
| 515.1625 | dHexHexHexAMe | 0.154 | < 0.154 | 96 | 45 | 18 | 4 |
|  |  |  | > 0.154 | 11 | 2 | 0 | 0 |
| 599.1955 | HexAHexNAcHexNAc | 0.189 | < 0.189 | 100 | 46 | 18 | 4 |
|  |  |  | > 0.189 | 7 | 1 | 0 | 0 |
| **Stroma region** | | | | | | | |
| 259.0135 | HexS | 0.0666 | < 0.0666 | 43 | 13 | 3 | 0 |
|  |  |  | > 0.0666 | 56 | 31 | 13 | 4 |
| 282.0290 | N-Acetylhexosamine sulfate | 20 | < 20 | 89 | 42 | 15 | 4 |
|  |  |  | > 20 | 10 | 2 | 1 | 0 |
| 396.1150 | HexAHexNAc | 1.42 | < 1.42 | 85 | 41 | 15 | 4 |
|  |  |  | > 1.42 | 14 | 3 | 1 | 0 |
| 401.9785 | N-Acetylhexosamine disulfate | 1.36 | < 1.36 | 92 | 43 | 16 | 4 |
|  |  |  | > 1.36 | 7 | 1 | 0 | 0 |
| 476.0725 | HexAHexNAcS | 0.0847 | < 0.0847 | 85 | 42 | 15 | 4 |
|  |  |  | > 0.0847 | 14 | 2 | 1 | 0 |

**Supplementary Table 1:** Cut off points and patients survival table of Kaplan-Meier analysis.
