# Supplementary material for: Native glycan fragments detected by MALDI mass spectrometry imaging are independent prognostic factors in pancreatic ductal adenocarcinoma
Source: EJNMMI Res. 2021 Dec 1;11:120. doi: 10.1186/s13550-021-00862-y (PMC8636555; doi:10.1186/s13550-021-00862-y)
Supplement: Supplementary file 3 — Additional file 3. Supplementary Table 2: Multivariate analysis of the prognosis significant glycan fragments with Union for International Cancer Control (UICC) stage. HR-hazard ratio; CI-confidence interval. [file 13550_2021_862_MOESM3_ESM.docx]

Supplementary Table 2:

| Glycan fragments | *P value (log p)* | HR (95% CI) |
| --- | --- | --- |
| Cancer cell region |  |  |
| HexS | 0.5998 | 0.78 (0.31-1.97) |
| dHexHexS | 0.6893 | 0.61 (0.05-7.07) |
| HexHexNAcAc | 0.9571 | 1.03 (0.30-3.57) |
| HexHexNAcS | 0.3749 | 0.41 (0.06-2.97) |
| dHexPenHexAc | **0.0467 *** | 0.57 (0.32-0.99) |
| KdoHexAHexA | 0.4577 | 2.53 (0.22-29.48) |
| HexHexHexNAcS | 0.7770 | 1.38 (0.15-12.79) |
| HexHexNAcHexNAcS | 0.7566 | 1.50 (0.12-19.22) |
| HexNAcHexPNeuAc | 0.9252 | 0.89 (0.08-10.23) |
| dHexHexHexSHexNAc | 0.4240 | 0.41 (0.05-3.60) |
| HexA | 0.2386 | 2.10 (0.61-7.19) |
| Chondroitin or Hyaluronan | 0.2884 | 1.77 (0.62-5.07) |
| Chondroitin sulfate | 0.9116 | 1.04 (0.53-2.03) |
| dHexHexHexAMe | **0.0386 *** | 2.58 (1.05-6.35) |
| HexAHexNAcHexNAc | 0.1276 | 0.35 (0.09-1.35) |
| UICC-Stage | **0.0303 *** | 1.45 (1.04, 2.02) |
| Stroma region |  |  |
| HexS | **0.0082 **** | 0.52 (0.32-0.84) |
| N-Acetylhexosamine sulfate | 0.2657 | 1.57 (0.71-3.50) |
| HexAHexNAc | 0.8024 | 0.90 (0.38-2.11) |
| N-Acetylhexosamine disulfate | 0.1271 | 2.14 (0.81-5.70) |
| HexANAcS | **0.0180 *** | 2.79 (1.19-6.53) |
| UICC-Stage | **0.0007 ***** | 1.76 (1.27-2.44) |

**Supplementary Table 2:** Multivariate analysis of the prognosis significant glycan fragments with Union for International Cancer Control (UICC) stage. HR-hazard ratio; CI-Confidence interval;
